# Establishing and Maintaining an Extensive Library of Patient-Derived Xenograft Models

**DOI:** 10.3389/fonc.2018.00019

**Published:** 2018-02-19

**Authors:** Marissa Mattar, Craig R. McCarthy, Amanda R. Kulick, Besnik Qeriqi, Sean Guzman, Elisa de Stanchina

**Affiliations:** ^1^Antitumor Assessment Core Facility, Molecular Pharmacology Program, Memorial Sloan Kettering Cancer Center, New York, NY, United States

**Keywords:** patient-derived xenograft, patient sample acquisition, patient-derived xenograft implantation techniques, patient-derived xenograft database, patient-derived xenograft propagation techniques

## Abstract

Patient-derived xenograft (PDX) models have recently emerged as a highly desirable platform in oncology and are expected to substantially broaden the way *in vivo* studies are designed and executed and to reshape drug discovery programs. However, acquisition of patient-derived samples, and propagation, annotation and distribution of PDXs are complex processes that require a high degree of coordination among clinic, surgery and laboratory personnel, and are fraught with challenges that are administrative, procedural and technical. Here, we examine in detail the major aspects of this complex process and relate our experience in establishing a PDX Core Laboratory within a large academic institution.

## Introduction

Over the past decade, patient-derived xenograft (PDX) models have come to represent an invaluable tool for a number of applications, including tumor genetics, biomarker discovery, the study of metastatic progression, the fate of circulating tumor cells (CTCs), the development of novel therapies for early, advanced, and drug-resistant tumors, and the implementation of cancer personalized therapy.

**Figure 1:**
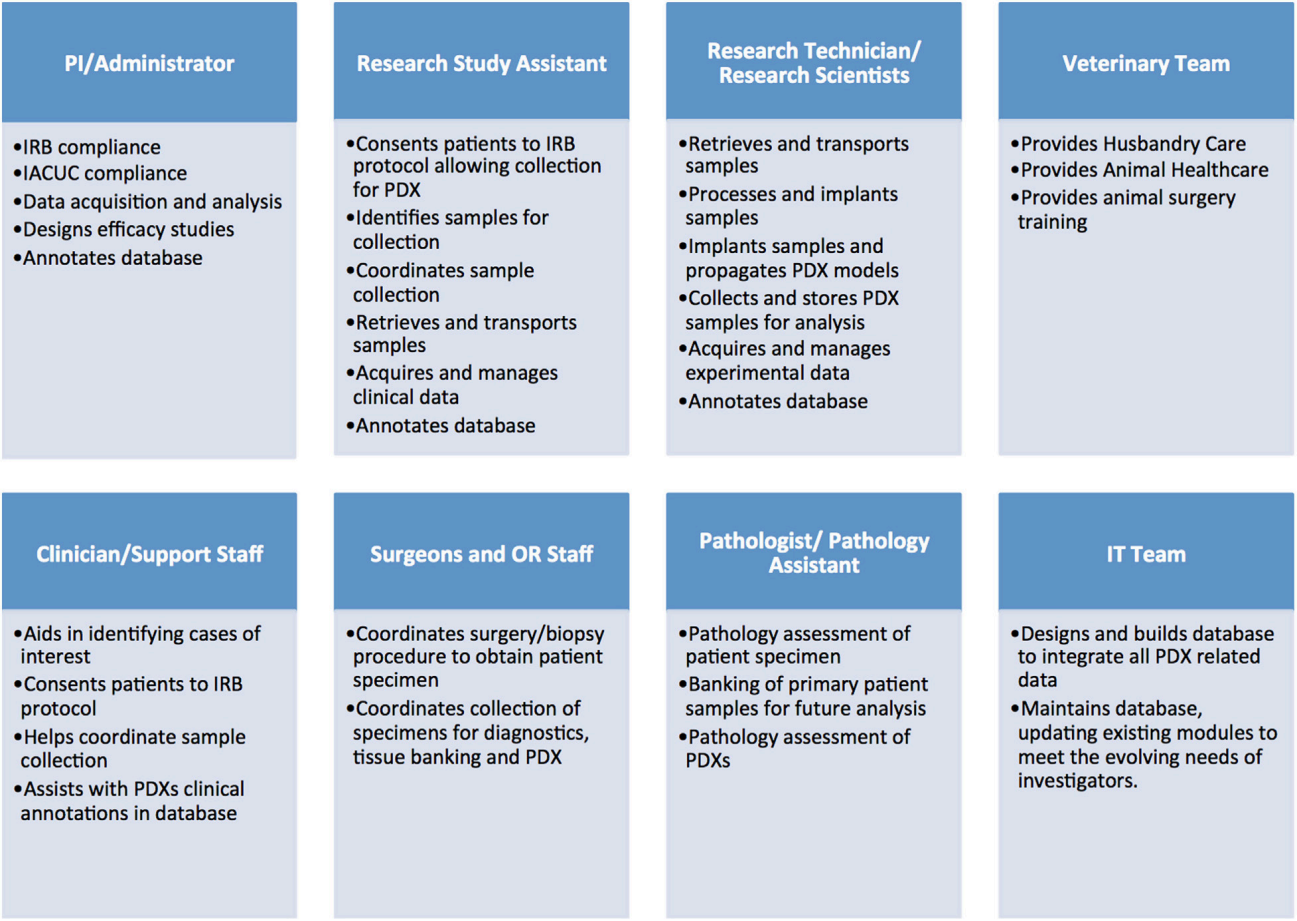
Administrative, clinical, and research personnel involved in establishing patient-derived xenograft (PDX) models.

Establishing libraries of PDX models is a complex and costly endeavor that requires a significant regulatory, administrative and laboratory infrastructure. Additionally, it necessitates the coordinated efforts of multiple teams, including: administrators, clinicians, surgeons, pathologists, and other medical personnel, research scientists, specialized lab and animal technicians, veterinarians, bioinformaticians, and IT support (Figure 1).

**Figure 2:**
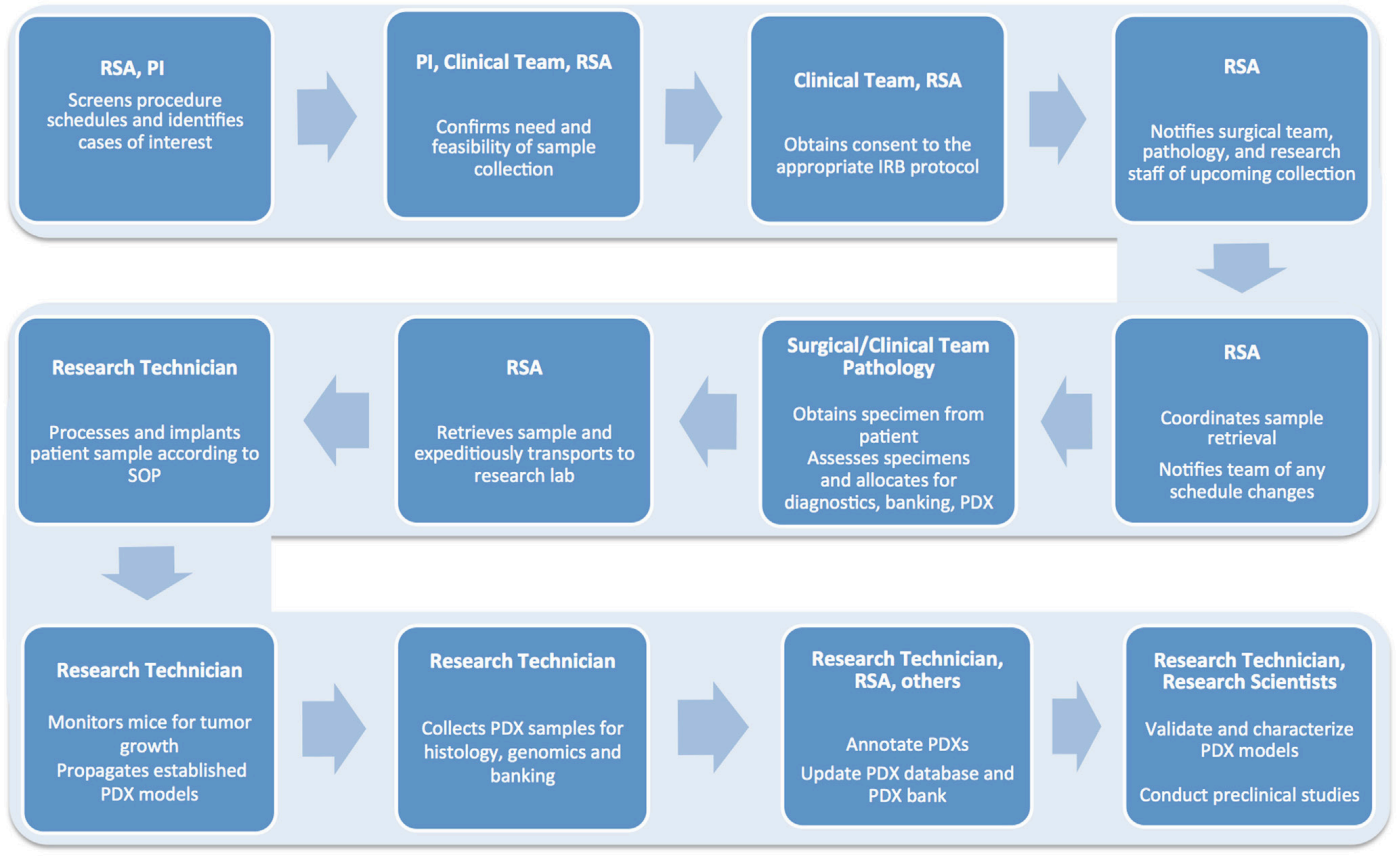
Flow chart detailing the numerous steps involved in the successful establishment, annotation, and propagation of novel patient-derived xenograft models.

A typical workflow for tumor sample collection, and subsequent PDX establishment and propagation, includes multiple tightly connected and time-sensitive steps (Figure 2). First, patients need to be selected according to specific criteria and consented to the correct Institutional Review Board (IRB) protocol. Next, surgery and pathology personnel need to be informed of the specimen request, so samples can be collected, examined, and transported to the research lab in a timely manner. Similarly, research staff must be alerted ahead of time to ensure the availability of reagents and personnel. When samples are received, they must be skillfully processed and implanted in mice or properly stored for subsequent analysis. Each resulting model needs to be molecularly and histologically characterized to allow for proper design and interpretation of future studies. Finally, sample annotation and rigorous documentation of each step has to be maintained throughout the entire process (1, 2).

In the sections below, we discuss the six main aspects of this process: regulatory and infrastructure needs; tumor sample screening and collection; sample processing, implantation and propagation; model validation and characterization; database annotation and management; and PDX use in preclinical studies.

## Regulatory, Hospital, and Laboratory Infrastructure Needs

### Requirements for Procurement of Human Tissues for Research

The procurement and biodistribution of human specimens and correlated patient health information is subject to stringent regulation (3).

In the US, tumor samples obtained for research-only purposes are subject to: (1) standard of medical practice: collection of specimens can only happen if the procedure is safe and feasible; (2) approval of a biospecimen collection and distribution protocol from the IRB (see also 45 CFR 46)^1^; (3) informed consent to sample collection under such approved IRB protocol; and (4) proper handling of identifiable patient health information as mandated by the federal Health Insurance Portability and Accountability Act (HIPAA)^2^ (4).

To address issues related to patient consent, we recommend consulting with the IRB office and instituting an umbrella IRB protocol, allowing for collection of surgical, fluid, and biopsy specimens from patients with a range of diseases for the purpose of establishing xenograft models and specifically requesting consent for germline and tumor genomic analysis as well as of sequencing data in central repositories. Importantly, efforts should be made to ensure patients are presented with the IRB consent paperwork well in advance of procedures, providing them ample time to discuss any concerns. This also allows the surgical team to properly prepare for the tissue collection. Additionally, samples should be labeled with a new unique ID immediately following collection to prevent mishandling of identifiable patient data.

### Occupational and Environmental Health Requirements for Working with Human Tissues

The manipulation of tumor samples and PDXs for research purposes involves potential exposure to hazards, which needs to be minimized. In the US, working with primary human tissue requires compliance with Occupational Safety and Health safety (OSHA) regulation^3^ and with the recommendations of the Institutional Biosafety Committee (IBC), which reviews the use of all laboratory hazards, including biologics. Tumors to be implanted in mice should be handled in accordance with Animal Biosafety Level 2 procedures, which require personnel to have adequate training for the usage of pathogenic agents and handling of infected animals, and mandate that physical containment equipment, such as biosafety cabinets, must be used whenever potentially infectious materials are handled (3). We recommend that IBC protocols, tissue sample handling procedures, and exposure control plans are reviewed frequently with all staff to ensure both compliance with regulation and a safe working environment.

### Requirements for Animal Use for the Development of PDX Models

The use of animals in research is strictly regulated. In the US, the Institutional Animal Care and Use Committee (IACUC) oversees all animal studies performed within an Institution, and ensures compliance with all relevant guidelines and federal regulations. We recommend to set out as soon as possible to draft a comprehensive IACUC protocol, with adequate rationale for the establishment and use of specific PDX models; step-by-step descriptions of all sample implantation procedures, tumor growth monitoring, and subsequent potential treatment protocols; adequate justification of animals needed and benefit-harm assessments (3).

### Other Legal Requirements

It is advisable to request the institution legal department to draft a blanket Material Transfer Agreement (MTA) so when a PDX model needs to be shared with outside collaborators, proper documentation can be easily compiled and no additional time is required to handle distribution of the model.

### Hospital and Laboratory Infrastructure

A robust hospital infrastructure, including specialized equipment and personnel, must be in place. Additionally, fully equipped research facilities with tissue culture, biobanking, and histology equipment should also be located nearby. The space where mice are housed is also crucial, as quality and efficiency of the vivarium and its husbandry operations and veterinary services directly and indirectly influence the quality and efficiency of the PDX program (5).

### Bioinformatics and IT Infrastructure

Annotation of PDX models requires access to clinical and sequencing databases to cull relevant medical and histopathology information and to retrieve and compare genomic patient and PDX data. While this large amount of information can be stored in multiple datasets, it is advisable to establish a dedicated, HIPAA-compliant PDX database, which will serve as an interface for collecting, storing, and tracking raw experimental data obtained from several sources, including tissue collection, biobanking, molecular and genomic analysis, and *in vivo* and *in vitro* experiments (5). This process is described in further detail below.

## Sample Screening and Collection

### Screening

Setting defined criteria for identification of potential tumor specimens for PDX generation is essential, as it is generally not feasible nor practical to collect samples from all patients undergoing a procedure. Most commonly, investigators are interested in establishing PDXs for disease subtypes for which treatment options do not exist or have been exhausted, or for which no current laboratory models exist. Additionally, some laboratories aim to generate large libraries of PDX models of the same tumor type to better understand heterogeneity and genomic characteristics within a given cancer subtype, or to establish models from the same patients throughout their disease course. This is especially useful in providing insights into tumor progression and mechanisms of resistance. Once selection criteria are in place, samples of interest can be identified by screening the schedules of upcoming surgical resections, endoscopies, biopsies, or fluids collections (blood, ascites, pleural and pericardial effusions, and bone marrow). While physicians and other support staff can provide invaluable assistance in pinpointing cases of interest, it may be prudent to assign a dedicated assistant to the task of identifying and coordinating the collection of desirable samples.

**Figure 3:**
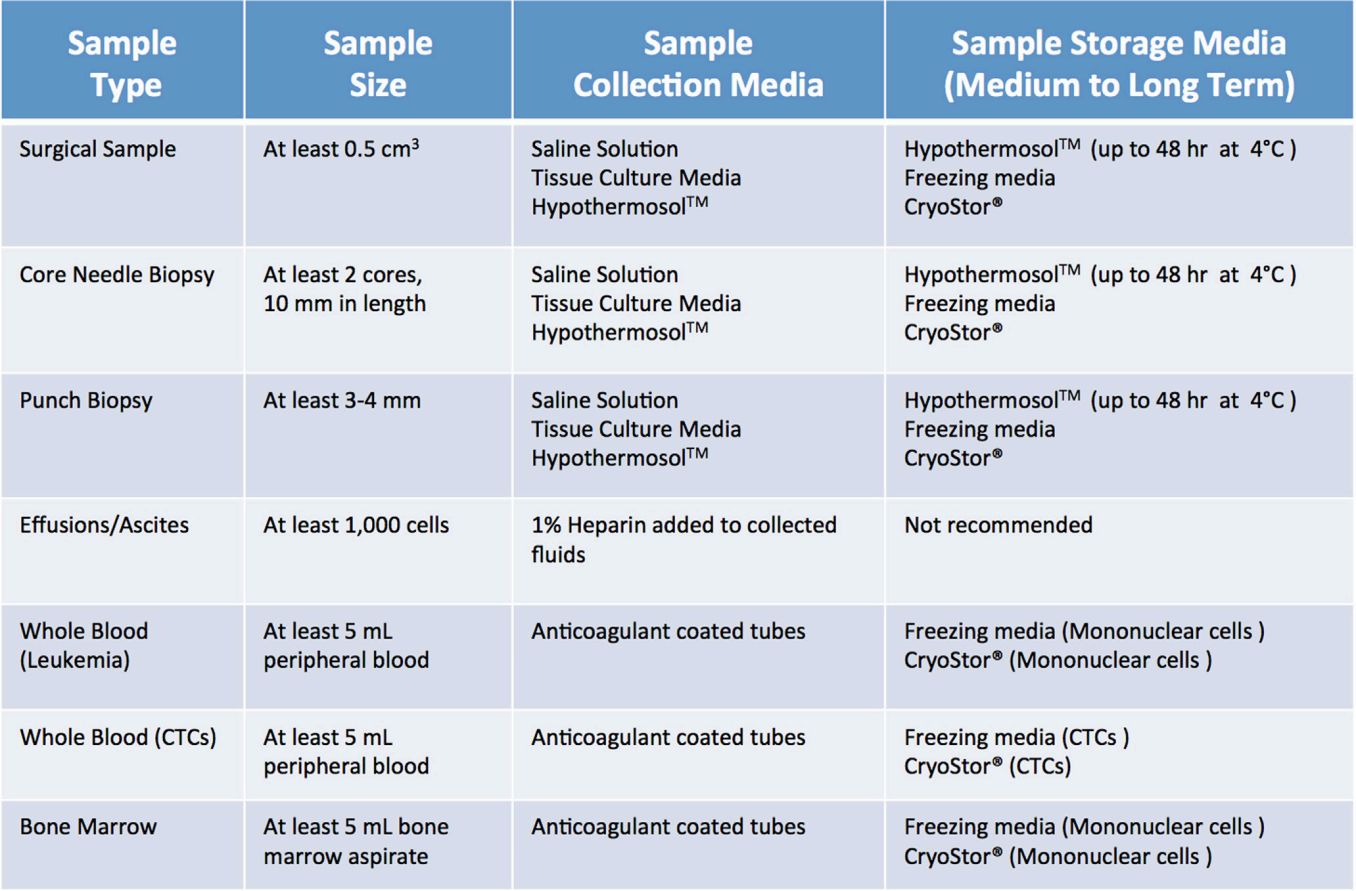
Recommended minimum sample size and storage media for clinical samples used for establishment of patient-derived xenograft models. Saline solution: 0.9% sodium chloride. Tissue culture media: DMEM or RPMI-1640, 10% fetal bovine serum (FBS) and antibiotics. Freezing media: 10% DMSO, at least 20% FBS in DMEM.

When screening, several factors need to be considered. Most importantly, a sample should be “flagged” only if the collection procedure is clinically safe and feasible, does not add any extra risk or burden to the patient, and does not interfere with the preservation of material for diagnostic purposes. Furthermore, minimum size requirements for tumor specimens need to be considered. As summarized in Figure 3 (1, 2), size requirements vary depending on procedure and sample type. In the case of core needle biopsies, the lesion should be at least 1.5–2 cm, to allow for collection of a minimum of 2 cores (10 mm in length) for PDX, in addition to the ones needed for diagnostic purposes. While core needle biopsies are the preferred method of non-invasive tissue acquisition, PDXs can be generated from limited material such as fine needle aspirates as well (1, 2, 6, 7). Punch biopsies, which produce a 3–4 mm cylindrical tissue core, are often used to obtain samples of cutaneous malignancies (8, 9). Similarly, samples of gastrointestinal tract cancers can be obtained *via* endoscopic procedures, which yield cores that are 2–3 mm^3^ in size. Since these tissue samples are smaller than their core needle biopsy counterparts, 4–6 cores should be collected for PDX implantation. When dealing with samples from surgical tumor resections, the lesion should be at least 2 cm. For hematological malignancies, a minimum of 5 mL of non-coagulated peripheral blood or bone marrow aspirate is required to ensure sufficient mononuclear cell (MNC) numbers, although engraftment rate of acute leukemia samples usually strongly correlates with blast percentage (1, 2, 10).

When screening, one should also be mindful of patients who may have infectious diseases that can pose a risk to research personnel and mice, or negatively impact tumor engraftment. In most cases, such samples should likely be excluded from collection. If the above criteria are met and the patient is consented to the appropriate IRB protocol, then a sample can be collected.

### Collection

Collecting biospecimens is a complex process requiring the coordinated efforts of multiple staff members including: clinical and surgical personnel, pathologists, research technicians, and veterinary staff (1, 2). To minimize tissue collection interference with other clinical and laboratory processes, timely communication with all members of the team is paramount. The surgeon or interventional radiologist and their support staff, along with pathologists and their diagnostic team, should be notified of each collection request as soon as possible, ideally days ahead of the scheduled procedure. Specifications regarding aspects of the PDX collection protocol, tissue size requirements, and sample preservation details should be clearly communicated to the entire team, along with relevant contact information for specimen pickup. Similarly, laboratory personnel should be alerted well in advance of each procedure. Details such as implantation modality (subcutaneous versus orthotopic), mouse strain and sex, hormone requirements (for estrogen or androgen-dependent tumors), along with expected date and time of sample retrieval should be circulated as soon as possible, to ensure the availability of personnel with the necessary expertise and adequate inventory of reagents, and immunocompromised mice. Importantly, a reminder should be sent out the day before the procedure to all personnel involved.

Of note, as it is not uncommon for multiple investigators to be interested in obtaining tumor samples from the same patient, it is highly recommended that a priority list for sample distribution is created and agreed upon by all parties involved ahead of the procedure to minimize any confusion and delay in sample retrieval.

Some institutions have created medical donation programs through which terminally ill patients can consent to samples being collected posthumously for research purposes. Samples collected for PDX from such procedures are truly invaluable as many patients, especially those who initially present with advanced disease, never undergo surgical procedures. This in turn leads to a huge deficit in reliable disease models for many aggressive tumor types. However, collecting samples for PDX from autopsies is an exceedingly complex process, which requires extra coordination as detailed in Mattar et al. (1, 2).

**Figure 4:**
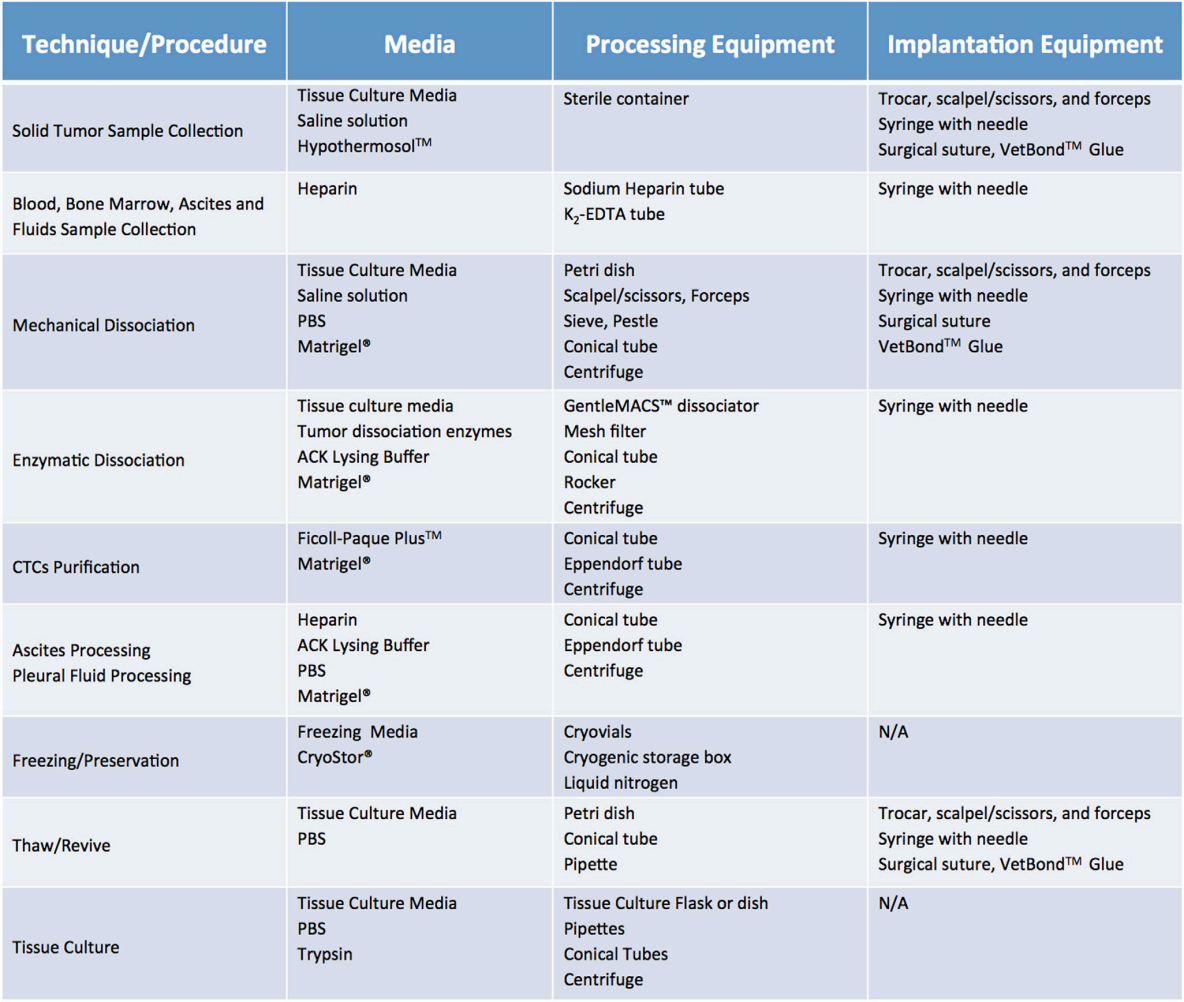
Recommended media and equipment needed to process clinical samples ahead of their implantation into mice. Requirements vary depending on tissue sample and chosen processing modality. Saline solution: 0.9% Sodium Chloride. Tissue Culture Media: DMEM or RPMI-1640, 10% fetal bovine serum (FBS) and antibiotics. Freezing media: 10% DMSO, at least 20% FBS in DMEM. Abbreviations: PBS, phosphate buffer saline; ACK lysis buffer, ammonium chloride potassium lysis buffer.

Once cases of interest have been identified, the prospective patient has signed informed consent paperwork, and the appropriate parties have been contacted, sample collection can take place. Although seemingly trivial, this process plays a relatively large role in the ultimate tumor engraftment success rate, so it must be handled with attention to detail as well as a sense of urgency. Caution must be taken at this step to reduce both warm ischemia, which encompasses the duration of the surgical procedure, and cold ischemia, defined as time elapsed between sample collection and implantation, as both inversely correlate with engraftment rate (11). Therefore, independent of sample type or collection method, it is paramount to keep samples on ice or in a refrigerated unit during all transportation and storage steps prior to implantation. In general, the optimal time limit from excision to implantation is 30 min to 1 h (11). In all circumstances, care should be taken to collect samples in aseptic conditions. Surgical and biopsy samples should be immediately placed in collection tubes prefilled with cold sterile media (Figure 4). Both saline (0.9% sodium chloride) and standard tissue culture media, such as RPMI or DMEM, are acceptable media for short-term storage. However, if samples cannot be implanted within an optimal time-frame, they should be preserved in media that have been shown to support cell viability over a prolonged period. In our experience, Hypothermosol™ helps preserve tumor cell viability for up to 48 h, and is the preferred medium for shipment of patient samples to and from other institutions, or for preservation of samples collected late at night or over the weekend (1, 2). In circumstances in which tissue implantation within 2 days of collection is not possible, tumor samples can be cryopreserved in freezing media and stored in liquid nitrogen in an effort to preserve tumor viability. However, freezing specimens ahead of their implantation is not recommended, as it has been shown to lead to lower take rates (11). Finally, for autopsy specimens, despite all technical and administrative complexities, efforts should be made to ensure retrieval of samples within 8–12 h from time of death, due to rapid decrease in cell viability post mortem (12).

Fluids, such as pleural and pericardial effusions, ascites, and bone marrow, are also considered a valuable source of tumor cells and tend to have higher engraftment rates than the corresponding solid tumor samples (13). Of note, fluid samples should be treated with heparin (1 mL heparin/liter of fluid) immediately after collection, to prevent clotting and facilitate sample processing. A summary of preferred collection media and procedures can be found in Figure 4.

## Sample Processing, Implantation, and Propagation

### Processing Solid Tumors

Processing and implantation methods can vary according to tumor type, size, and available resources. However, it is crucial that all specimens are processed and implanted employing sterile techniques to prevent contamination, and are handled in accordance with institutional policies pertaining to potentially hazardous materials.

Surgical tumor samples are typically quite heterogenous, potentially containing cysts and regions of necrosis along with areas of normal tissue, all of which need to be removed to increase chances of tumor uptake (14, 15). Surgeons and pathologists may be able to assist in the initial processing of the bulk sample, but most of the debriding is usually carried out by the technicians tasked with sample implantation, who should be adept at recognizing and isolating viable tumor material from adjacent normal and necrotic tissue. This is not a trivial task and requires hands-on training with multiple tumor and tissue types. Viable tumor regions, composed of both tumor cells and stromal components, will usually appear to have a different color, morphology, shape, and consistency, often looking less opaque and firmer than the neighboring healthy tissue. Necrotic tissue is most commonly found at the center of large tumors; however, its appearance and texture can be quite variable, ranging from opaque and white to very dark in color, and can be brittle, hard, or liquid. Stromal connective tissue is usually recognizable because of its translucent, stretchable nature, while adipose tissue usually has a soft consistency and can be easily peeled away with a blunt or sharp instrument. Care should also be taken to remove any foreign materials such as sutures, staples, gauze, and hair.

During the debriding process, surgical samples are usually placed in Petri dishes and submerged in cold saline or tissue culture media. Then, normal, clotted, and necrotic tissues are excised with sterile forceps, scalpels, and scissors. The clean tumor sample is then transferred to a new dish and sectioned into smaller fragments (16).

**Figure 5:**
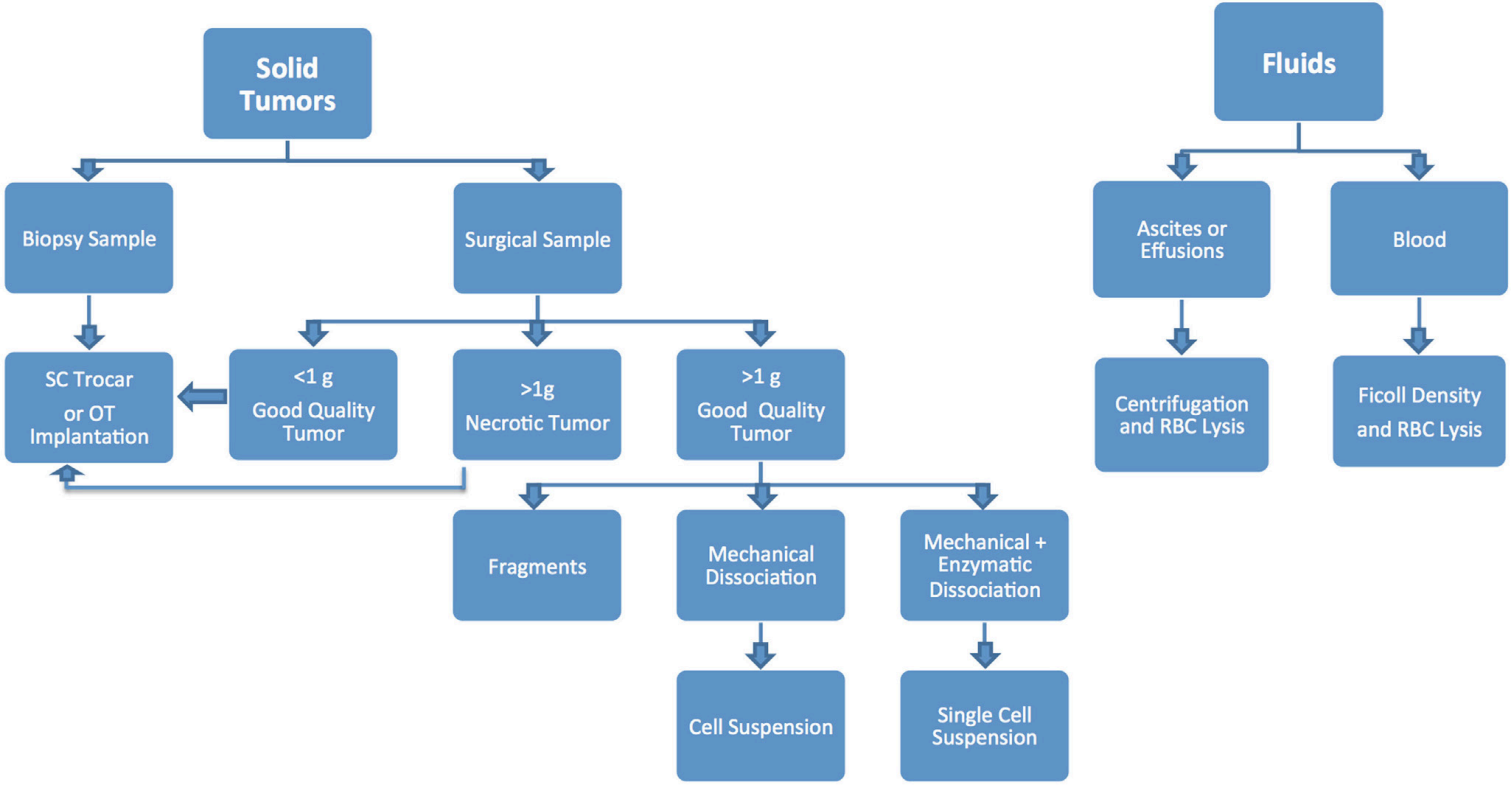
Techniques recommended for processing clinical samples based on sample type and size.

Different modalities can be used to implant tumor specimens, mainly based on how much material is available and preferred implantation method (Figure 5). Small surgical samples (i.e., <1 g) or biopsies are usually implanted without additional handling, as dissociation and processing can drastically reduce the number of viable tumor cells, resulting in poor take rate (1, 2). Larger samples can be sectioned into smaller fragments (~2 mm), and implanted as such, or can be further dissociated to obtain a homogeneous cell suspension.

Mechanical dissociation can be accomplished by repetitive pipetting or triturating the tumor samples by pressing it with a pestle through a 600 µm sieve and into a sterile beaker. The cell suspension obtained using this technique is usually composed of both single cells and cell aggregates, so it is not suitable for intravenous (IV) injection, but can be mixed with matrigel and injected using a 22, 20 or 18 G needle subcutaneously (SC) (1, 2). Tumor samples can be further processed *via* enzymatic digestion with collagenase and hyaluronidase and trypsin/EDTA to generate a single cell suspension, which can then be injected IV or orthotopically (OT). Tumor dissociation kits (such as gentleMACS™), which include cocktails of lysing enzymes to be used along with a mechanical dissociator, allow for efficient, semi-automated dissociation of tissues into single-cell suspensions or thorough homogenates (1, 2).

**Figure 6:**
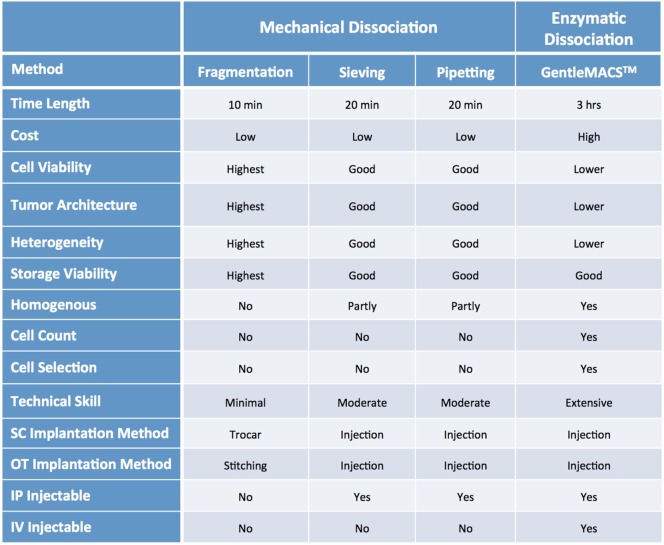
Characteristics of common sample processing and implantation methods.

For many solid tumor types, the implantation of tumor fragments is favored over the implantation of samples dissociated into single cells, as it can be accomplished quickly and efficiently, with little impact on cell viability. Moreover, it minimizes the loss of stromal components and other tumor architectural features that may be essential for engraftment. By contrast, the more aggressive processing techniques can subject the tumor cells to harsh and stressful conditions, resulting in decreased cell viability and lower tumor take rates, especially for heterogenous tumor types (1, 2, 17–20). On the other hand, methods to produce single cell suspensions present with several advantages: they allow for easy assessment of tumor cell viability and for the isolation of specific tumor cell subpopulations prior to cell implantation. Additionally single cell suspensions can be easily injected both SC and OT, and can be cultured *in vitro* to generate novel cell lines (Figure 6).

### Processing Liquid Samples

Blood, pleural or pericardial effusions, ascites, and other fluid samples are processed differently than solid tumors. Blood samples and bone marrow aspirates from patients with hematological malignancies can be processed for PDX implantation *via* the isolation of MNCs by Ficoll density centrifugation followed by red blood cells (RBCs) lysis (1, 2). CTCs, which are commonly present in blood from patients with highly metastatic solid tumors, can be isolated from peripheral blood as described above, with the addition of special enrichment buffers to assist with the selection of tumor cells (21, 22). Both CTCs and tumor cells derived from hematological malignancies are usually implanted in mice *via* intravenous (IV) injections. When processing samples derived from effusion and ascites collection procedures, tumor cells can be isolated by centrifugation of the samples and repeated treatment of the resulting cell pellet with RBCs lysis buffer (ACK). The ensuing cell population can then be injected either SC or OT (13, 23, 24).

### Implantation Techniques

In addition to the sample preparation, the method and site chosen for sample implantation largely contributes to the successful engraftment of a PDX model. Tumor samples can be implanted either orthotopically into their anatomical sites of origin, or heterotopically, usually SC in the flank of immunocompromised mice. When implanting tumor fragments SC, a 10 gauge trochar should be used, whereas fine needle biopsy samples may be implanted with a smaller gauge (13–16 G) trochar. In such cases, all biopsy cores (usually 2–4) are placed in the same trochar and implanted together. If there is enough quality material, tissue can be implanted SC on both flanks of the recipient mouse. In these cases, care should be taken to implant similar amounts of tissue on both flanks, to ensure similar growth rates of the ensuing PDXs.

Logistically, heterotopic implantations are favored over orthotopic ones, as they are technically simpler, faster, and have a very low likelihood of procedural complications. Additionally, the growth of subcutaneously implanted tumors is easy to monitor and measure, making SC PDXs ideal models for exploratory studies identifying cytotoxic agents and early stage preclinical studies testing the efficacy of novel compounds. However, heterotopic models seldom metastasize (17, 25), and therefore may not accurately mimic the behavior of the human tumor from which they are derived.

On the other hand, orthotopic models are considered more physiologically relevant, are often highly metastatic, and are generally considered better predictors of clinical response and sensitivity to therapies whose mode of action may be modulated by the tumor microenvironment (14, 26, 27). Despite the obvious advantages, the use of orthotopic models is limited by a number of technical challenges. Orthotopic implantations are costly and labor intensive, may require the use of specialized equipment, need to be performed by highly skilled technicians, and can lead to post-surgical complications. Moreover, accurate tracking of tumor volumes and response to therapy in orthotopic models is quite cumbersome and usually requires the use of small animal imaging modalities, such as ultrasound, computed tomography, magnetic resonance imaging, and positron-emission tomography, which may not be readily available (28). While orthotopic implantation procedures vary depending on the organ and tumor type, it is important to note all orthotopic surgeries should be performed by trained personnel, using aseptic techniques and accepted veterinary practices for animal anesthesia, pain relief and post-operative care. Detailed descriptions of implantation procedures for the most common organs can be found in Uthamanthil et al. (29), and in the many articles referenced within its chapters.

### Tumor Engraftment

The successful generation of a PDX is reliant on the innate properties of the primary tumor sample and methods of sample handling, processing, implantation, and chosen mouse strain (11). Pathological attributes implicated in tumor engraftment include percentage of tumor cells, tumor subtype, metastatic potential, tumor stage and grade, hormone dependence, tumor location, and tumor quality. The engraftment rate based on histological subtype varies widely from under 20% to over 80% (15). As one would expect, samples with higher proportions of tumor cells are more likely to engraft (30). Additionally, specimens collected from metastatic sites have higher engraftment rates than samples from primary tumors (24). Further highlighting this point, PDXs established from malignant fluid samples (ascites, pericardial or pleural effusion) are more likely to engraft than the corresponding solid tumor samples, due to their highly cellular nature (13). To this end, a definitive correlation has been made between tumor engraftment in PDXs and poorer clinical outcomes in breast, lung, bladder, and colorectal cancer patients (31–35). In general, take rates are higher in more immunodeficient strains; therefore, NSG (NOD/SCID/IL2Rγ) mice are preferred over less immunocompromised strains such as athymic nu/nu or NOD/SCID mice (36–38). More recently, cotransplantation of human immune or stromal cells with the tumor specimen has also been employed to better recapitulate tumor progression (39–42).

**Figure 7:**
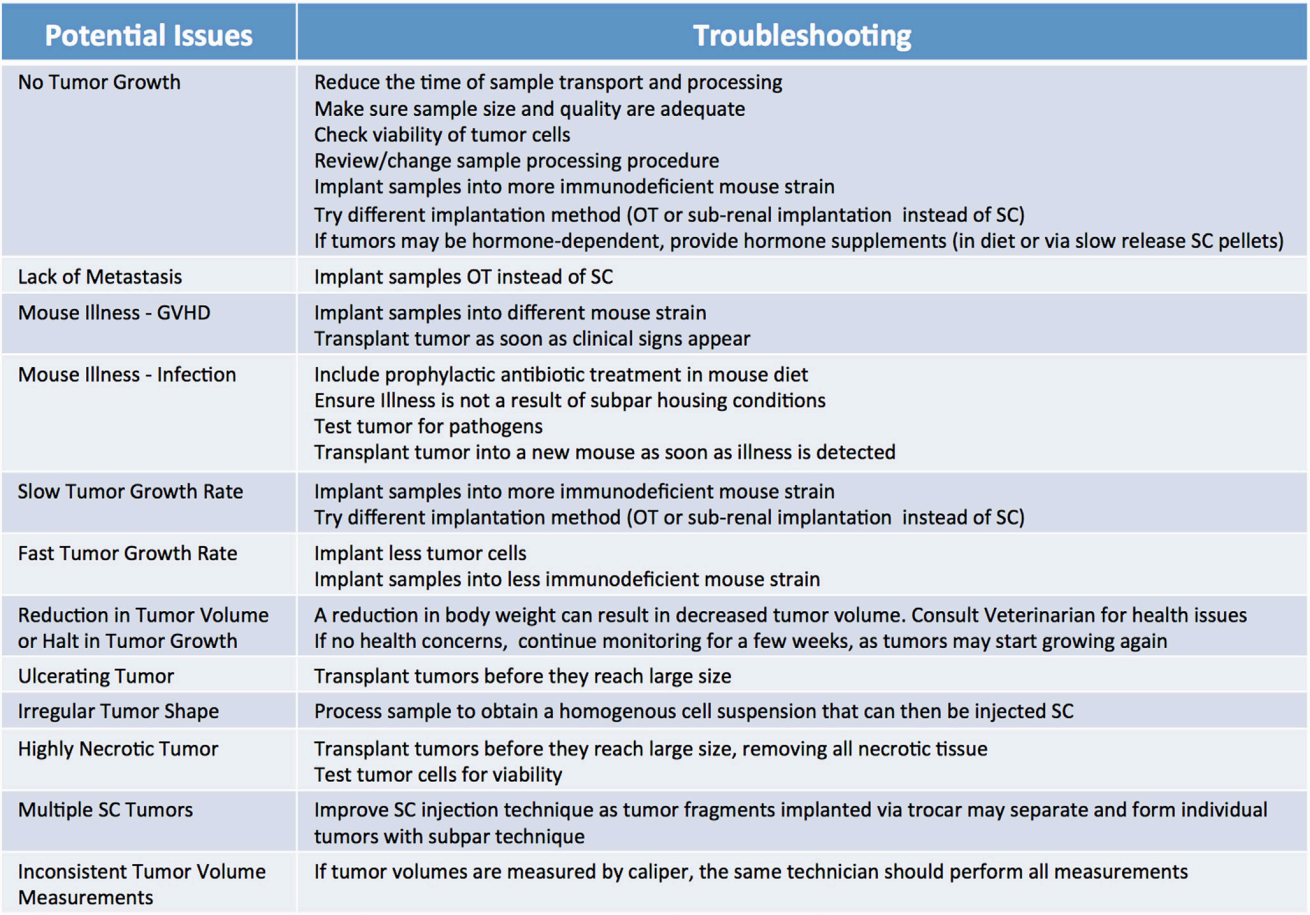
Factors influencing patient-derived xenograft take rate. Potential issues and corresponding suggestions for improvement. GVHD, graft versus host disease; OT, orthotopic; SC, subcutaneous.

Because of their compromised immune system, NSG mice are extremely vulnerable to infections; therefore, it is advisable to supplement their diets with antibiotics, such as Sulfatrim, for the entire duration of the study. Of note, the use of non-sterile techniques during collection, processing, and implantation can result in the introduction of pathogens that may not only inhibit the ability of the tumor to engraft, but may also compromise the health of the entire mouse cohort. Thus, veterinarians should be immediately consulted if transplanted mice exhibit signs of illness and their tumors should be tested for pathogens. It is important to note engraftment may induce an immune response against the murine host system (43), often referred to as graft versus host disease (GVHD). While not entirely preventable, the onset of GVHD can be delayed somewhat by the use of a strain of NSG mice lacking MHC-I (44). Furthermore, xenografted immunocompromised mice are vulnerable to spontaneous lymphomagenesis, and are susceptible to EBV transformed cells from the human donor (45–47). This phenomenon can be detected by routine phenotyping of primary tumors and xenografts with human lymphocytic markers (45, 47). Some murine viruses which are difficult to eradicate, such as lactate dehydrogenase–elevating virus (LDEV), can also interfere with the generation and propagation of tumor models (48). LDEV is most commonly transmitted *via* contaminated biological materials, such as Matrigel, thus ensuring purity of all biological materials is paramount for preventing infections. LDEV can be removed from a contaminated sample by either passaging the tumor in a rat or using a FACs based tumor cell purification protocol (49, 50). A list of the most commonly observed issues leading to poor engraftment rate, and recommendations on how to troubleshoot them, are summarized in Figure 7.

### Tumor Monitoring, Propagation, and Preservation

The growth rate of PDXs is highly variable, with tumors initially appearing in as little as several weeks or in excess of eight months (51), so it is recommended to monitor implanted mice for PDX growth for a minimum of 6 months before euthanizing them. Subcutaneously implanted tumors should be monitored at least once a week and tumor volumes measured with a digital caliper using the formula: $\left(\frac{\pi }{6}\right)\times \text{Length}\times {\text{Width}}^{\text{2}}$. To prevent tumor necrosis and skin ulcerations, it is advisable to limit tumor size to 500–1,000 mm^3^. At this point, tumors should be collected and can be transplanted and expanded into recipient animals (see below). Of note, if a mouse shows signs of sickness or an ulcerated tumor begins shrinking, then the tumor should be transplanted as soon as possible independently of its size, as to prevent loss of the model. As previously indicated, tumor progression in orthotopic and metastatic models is usually monitored using different imaging modalities, while in the case of hematologic models, progression of disease can be assessed by peripheral blood and bone marrow analysis.

**Figure 8:**
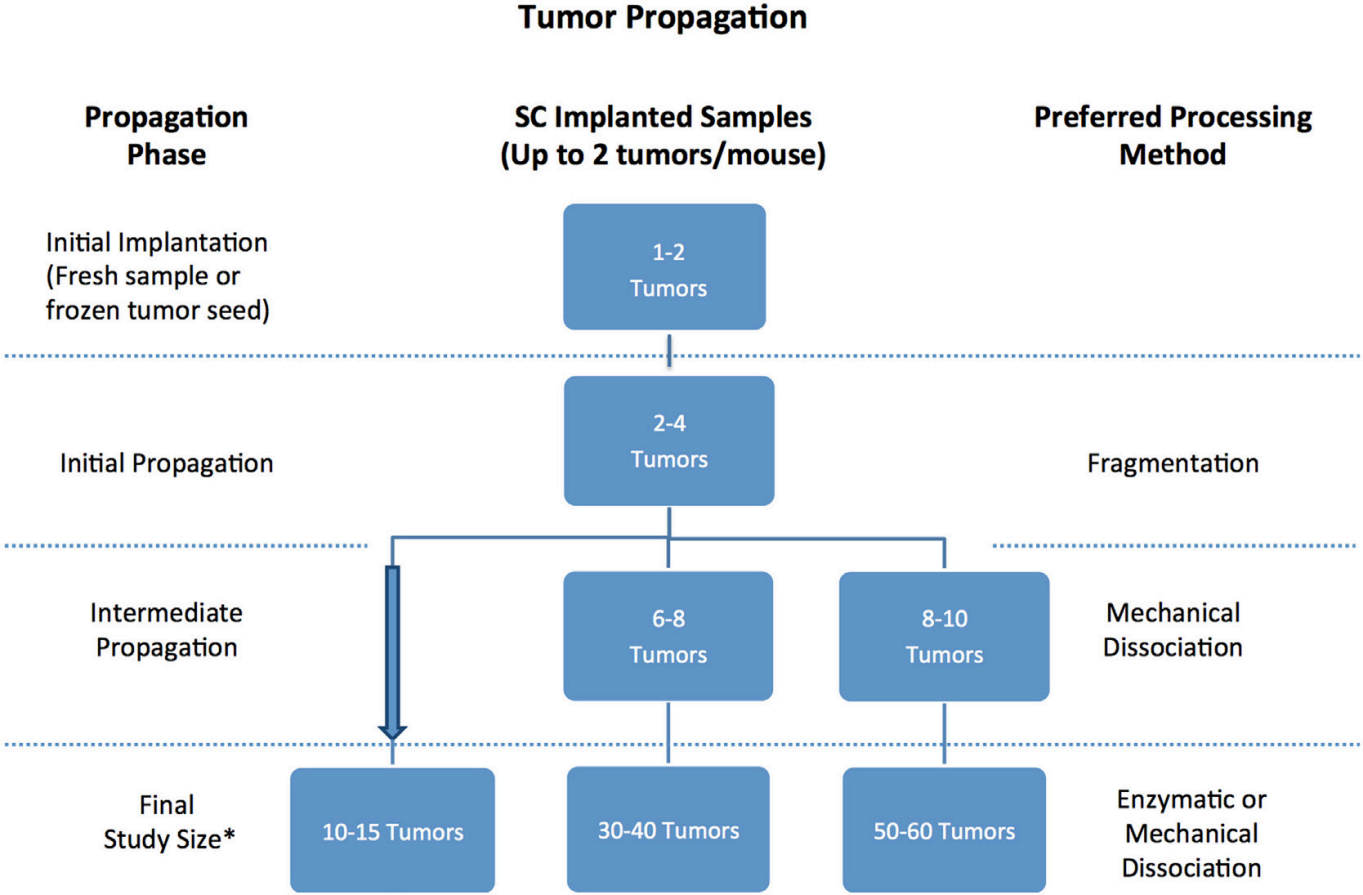
Recommended number of tumor samples needed for patient-derived xenograft propagation based on propagation scope and tissue processing preferences. *Always implant 20% more mice to account for variability in tumor volume.

Once a patient tumor first grows *in vivo* (referred to as passage 0), it can usually be retransplanted successfully and serially transplanted over several additional passages (referred to as p1 …to pN) (Figure 8). With each passage, care should be taken to remove stroma and necrotic tissue and isolate the viable tumor material for transplantation or preservation. PDXs are often maintained in a few mice until passage 3, before tumors are either viably frozen for future use or expanded into larger cohorts of mice for preclinical studies. When expanding for an efficacy study, it is recommended to implant the tumors single flank and to implant tumors into 20% more mice than the desired study cohort number to mitigate any variations in tumor volumes and engraftment rates in individual mice. Each PDX model exhibits unique morphologic and histologic characteristics, so it is important to keep detailed notes on tumor growth rate and tissue appearance, and to define an optimal processing strategy for each PDX, so that samples are processed and passaged consistently. It has been reported that PDXs tend to increasingly grow faster as they are serially transplanted, likely due to progressive substitution of human stroma with murine stroma (51, 52). To monitor for possible genetic or phenotypic drift, it is recommended to collect material for both sequencing and histologic analysis each time a tumor is transplanted (1, 2).

In order to create a PDX library, viably frozen tumor samples (“seeds”) must be harvested at each passage. Early passage seeds should be preferentially banked, and records should be kept indicating how many times a PDX model has been passaged. In general, tumors are resected and processed for freezing when they are of adequate size (i.e., 500–1,000 mm^3^) to produce enough material for several vials. However, preferred tumor size varies with each model, as ulcerations and necrosis can occur even in small tumors. Once a tumor is harvested, any non-tumor and necrotic tissue should be removed, and the viable tumor should either be cut into small fragments (~10 mm^3^) that can be immediately placed in prechilled cryovials containing appropriate freezing media or processed to obtain a single cell suspension. In this case, upon determination of cell number and viability, cells are mixed with freezing media and distributed into cryovials, ideally at a concentration of 1–10 million cells/mL, as cells frozen at a lower or higher density are often less viable. Tubes, appropriately labeled, should then be placed in a −80°C freezer in specialized containers designed to achieve an optimal cooling rate (~−1°C/min) for cell preservation. By the next day, samples should be transferred into a liquid nitrogen tank, taking care to log their exact location into the biobank database to facilitate future retrieval.

Frozen PDX seeds can be thawed, similarly to cell lines, by immersing the cryovials in a clean 37°C water bath for a few minutes. Once thawed, samples are transferred into conical tubes along with PBS. Cell suspensions are then centrifuged at 1,200 rpm for several minutes. After a second wash with PBS, PDX tumor cells or fragments are ready to be implanted, as described above. It is important to note that PDX samples implanted from frozen seeds take longer to grow than when they are propagated using fresh tissue (53).

## Sample Characterization and Validation

### Genomic Profiling

Thorough genomic/molecular analysis of PDXs and the corresponding human tumor from which they are derived is an integral component of proper PDX characterization. First, it is important to verify that the PDX model has a genomic profile consistent with that of the human specimen it was derived from. Additionally, analysis of sequencing data can permit the study of the evolutionary trajectory of recurrent tumors; help define the biologic basis of early treatment failure or the development of acquired resistance; and inform study design when PDX models are used in preclinical efficacy studies.

Genomic sequencing of PDX samples should be done in compliance with all institutional policies and local laws governing patient privacy to minimize possible inappropriate use of these data. Of importance, in addition to tumor material, it is critical to obtain normal tissue from the patient, so germline and somatic variants can be distinguished. Normal tissue can be obtained from a variety of tissue types. For solid malignancies, blood may be the most accessible source, since it can be collected either at time of surgery or during follow-up, and stored at −20°C until needed. Alternatively, institutional tissue bank services may be able to provide fresh frozen or formalin fixed material (54).

A unique problem with genomic characterization of PDX models is the presence of contaminating mouse DNA, arising from stromal cells residing in the tumor itself, along with trace amounts of other mouse tissues (skin, hair, etc.) that may be excised along with the tumor material. This problem can be addressed using a combination of cellular purification techniques and bioinformatics procedures to deplete the mouse genome reads. A detailed review of such techniques can be found in Poirier (54).

Since genomic data for both patient and corresponding PDX samples can be captured through multiple platforms (whole-genome, exome, and transcriptome sequencing; epigenomics and metagenomics; and capture-based targeted sequencing assays such as MSK-IMPACT) (55), it is important to build a database to consolidate data and facilitate comparative cross-species and longitudinal analyses (5).

### Histology Review

Tissue from both newly established and from serially transplanted PDX models should be routinely processed for H&E staining and reviewed by a board certified pathologist, to ensure that the lesions indeed correspond to the expected tumor type. This is of great importance, because lesions observed at the site of implantation may be of an inflammatory nature (granulomas or abscesses), or result from unrelated tumors of murine or human origin. While inflammatory lesions are usually easily identified, tumors must be more carefully analyzed. As mentioned above, most murine tumors are usually lymphomas, although fusiform cell sarcomas and mammary gland tumors have also been reported (46, 56). Additionally, human lymphomas developing at the engraftment site of non-lymphoid tumors are usually derived from lymphocytes present in the engrafted specimens, which happened to be infected with EBV. These B Lymphocytes can be efficiently eliminated in an immunocompetent system; however, malignant B cells can develop in immunocompromised mice (45–47). When a new PDX is established, it should be characterized both in terms of its histological type and differentiation (56). While tumor histological subtype is usually well-preserved in PDXs (57), some tumors may become less differentiated over time, and specific morphological details may change during serial passaging (58). Independent PDXs generated from the same specimen may not be identical due to intrinsic intra-tumor heterogeneity. Thus, when comparing histology of human specimens to their PDXs, it is important to note whether the global pattern and histology of the samples match, and additional IHC staining may be required to assess whether specific biomarkers present in the human specimen are also preserved in the PDX. In all cases, it is recommended that PDXs undergo histological review every few passages, and that high resolution microphotographs of H&E and IHC slides from human and PDX tumors should be preserved, along with the pathologist review for each newly established model, for easy review. Detailed information on how to better preserve PDX samples for histologic analysis, IHC staining techniques and recommended immunolabeling can be found in Fontaine et al. (56).

### Biobanking

Ample space should be allocated for storage of PDX-related specimens, including viable tumor tissue (“seeds”), samples set aside for genomic analysis (DNA/RNA from PDX tumor tissue, patient tumor, and normal tissues), and for histology (paraffin blocks, frozen tissues, slides). Ideally, a barcoding system for sample inventory and tracking should be set in place for easy cataloging and retrieval of archived samples (5). Additionally, rigorous standards to continually reaffirm the identity, viability, and purity of the PDXs models and any cell line that may be derived from them must be employed. To accomplish this, Short Tandem Repeat (STR) Analysis can be used to fingerprint PDX tumor lines and to reconfirm their identity (59). To guard against sample misidentification during the latency period after initial engraftment, STR analysis can be performed on DNA from patient blood samples collected and banked at the time of tissue donation. It is also important to test all established PDX models for murine infectious agents, as many of the immunodeficient mouse strains commonly used to propagate human PDX tumor tissues are susceptible to opportunistic pathogens (24, 60).

## PDX Database Annotation and Management

Managing the variety of data associated with preclinical models, especially those derived from patients samples, is an arduous task. Most commonly employed methods for PDX model annotations, such as Excel spreadsheets, are quite primitive, and lack the functionality to capture the multitude of complex and diverse data pertaining to these models in a meaningful way. In order to address the issues associated with data capture and management, it is recommended to develop a comprehensive yet dynamic HIPAA-compliant database. This centralized database should serve as an interface between the multiple parties within the institution involved in the generation and exploitation of these preclinical models, and would allow researchers to accomplish several goals including: management of raw data, tracking of experimental data, and access to clinical and genomic annotation of patient and PDX samples. Although an expensive effort, both in terms of time and cost commitment, a functional centralized PDX database ultimately allows for optimized and standardized data acquisition, straightforward data analysis, and easy selection of PDX models of interest within a large library. This in turn facilitates the synergistic interaction of laboratory investigators and clinicians to advance translational research.

Key features of the centralized PDX database should include the clinical annotation of patient and PDX samples, and collection of genomic and histology data, along with any *in vitro, ex vivo* and preclinical data associated with a specific PDX model, and biobank information. If possible, most of the data input fields should be predetermined, and free text options minimized. These guidelines not only enforce standardized data recording, but also allow for records to be easily searchable (5).

### Patient Sample Annotation

**Figure 9:**
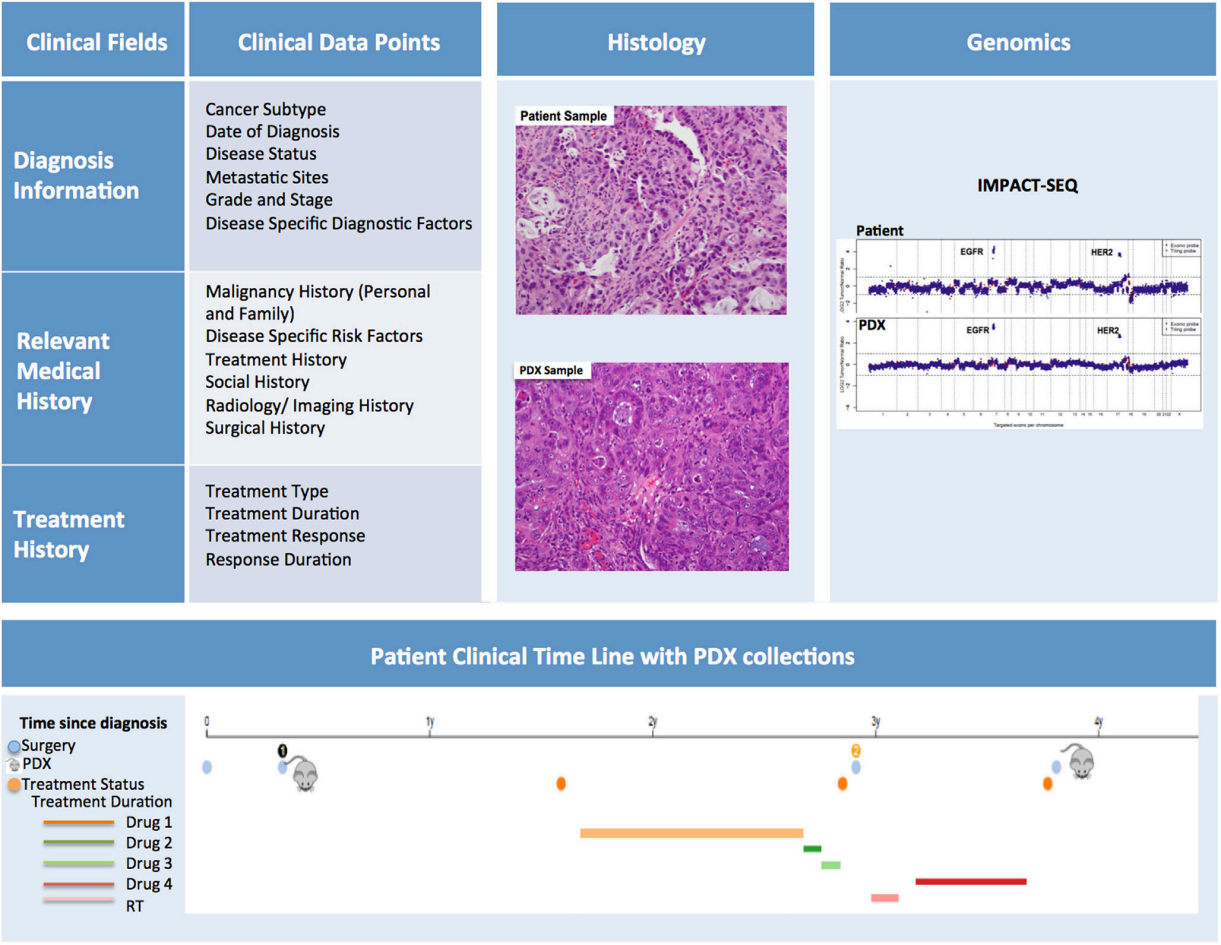
Patient-derived xenograft (PDX) database annotation should include clinical information pertinent to diagnosis, relevant medical and treatment history for each patient. Additionally data on histopathology and genomics characterization of both patient and PDX samples should be readily available. Finally, a timeline aligning patient treatment and time of PDX model generation can be very useful, especially for cases in which multiple PDXs have been generated from the same patient.

In order to maximize the utility of PDX models, the patient samples should be thoroughly annotated with pertinent clinical information (Figure 9). The data capture should commence as soon as a patient is enrolled for sample collection. At this time, basic demographic and diagnostic information along with relevant medical history can be entered into the database.

#### Diagnostic Information

Diagnostic information should include date of diagnosis, disease status (i.e., *de novo*, refractory, recurrent, etc.), evidence (if any) of metastatic sites, information pertaining to subtype, grade and stage, and mutational status. It is helpful to note the difference between the staging/grading at diagnosis and the staging/grading at time of sample collection. Disease specific diagnostic factors, such as Gleason scores for prostate cancer, should also be included in this section.

#### Relevant Medical History

The Relevant Medical History section should include personal and family history of malignancies, history of common and disease-specific risk factors (i.e., smoking history, H. pylori infection, reproductive history), and any other notable risk factors. In addition, a detailed history of the patient’s cancer treatment must be incorporated into the database. This section should include information about treatment type, duration, response, and duration of response. Moreover, the database should include selected information pertaining to the patient’s imaging and surgical history, along with relevant follow-up annotations. Updates on patient treatments occurring after the collection of the samples for PDX should also be included as they can be very informative, particularly when planning preclinical studies to assess efficacy of targeted therapies compared to standard of care. While the clinical information for in-house samples can easily be retrieved from the hospital medical records by authorized individuals, clinically annotating PDXs generated from outside samples presents more obstacles. For these cases, a minimal clinical dataset should be collected at the time the sample is received for implantation.

### PDX Sample Annotation

**Figure 10:**
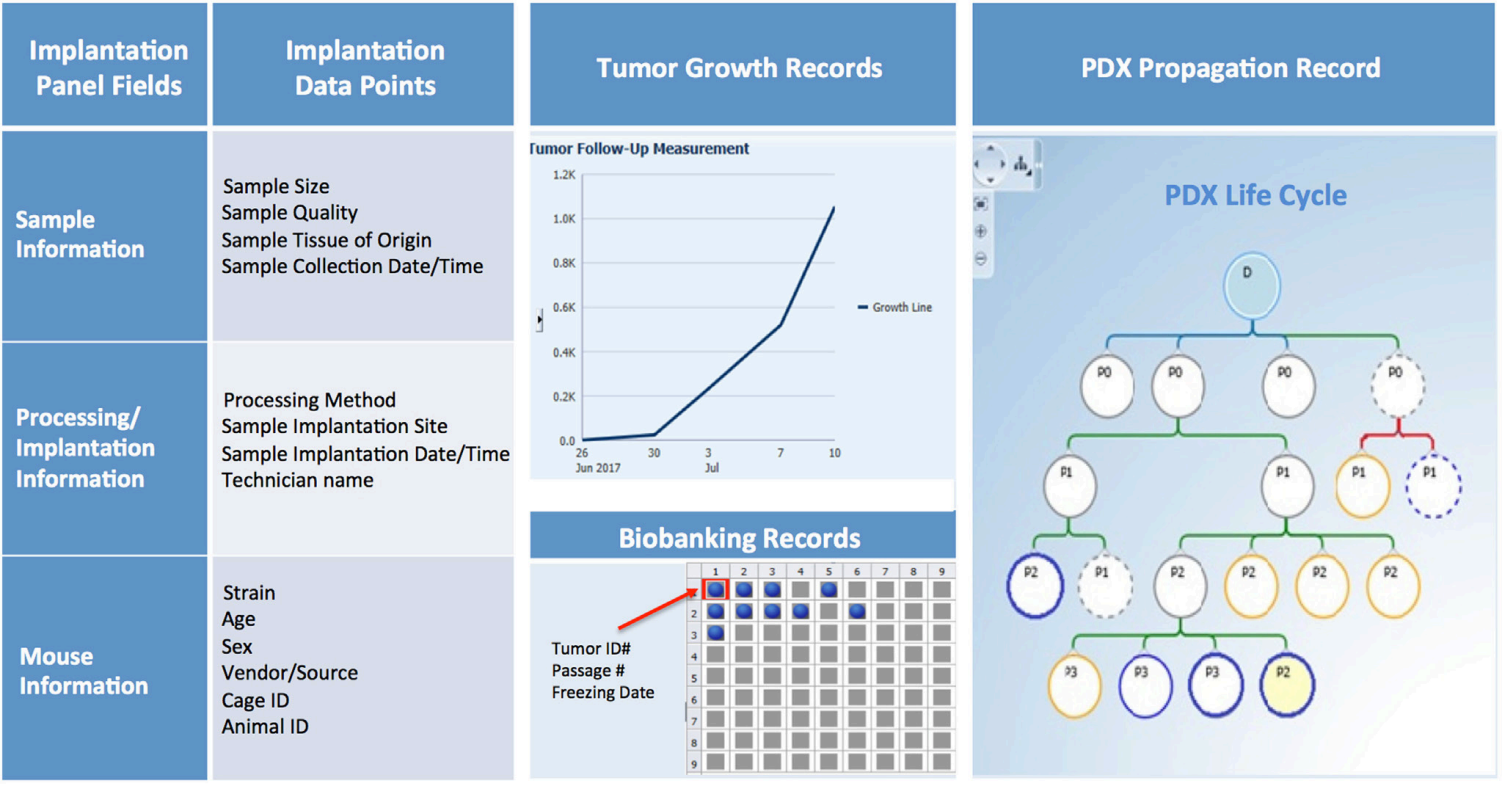
PDX database annotation should include information about sample processing and implantation, and records of PDX growth, propagation, and banking.

Patient-derived xenograft sample annotation should be as thorough as the annotations for the corresponding clinical samples. Records should include information on sample collection and implantation, PDX growth rate and other characteristics, and detailed documentation of all banked samples (Figure 10).

#### Collection and Implantation History

Collection and implantation history should include information on date and time of sample collection and its primary or metastatic classification, in addition to details on type of procedure (surgery, biopsy, etc.), medical team, and time elapsed from sample retrieval to implantation in mice. Information on size and quality of the sample, implantation site, and methodology should also be recorded and can be useful when troubleshooting factors that may contribute to poor engraftment (Figure 10).

#### In-Life History

For each PDX model, basic information such as mouse strain, sex, age, source, and any non-standard husbandry conditions (i.e., antibiotic diet, etc.) should be entered in predetermined fields, along with animal and cage ID, and housing location. Other records should include tumor volume measurements over time, clinical observations, imaging, pathology data, and the date of animal sacrifice and tumor transplant (Figure 10).

#### Banking History

Samples may be viably frozen for future transplantation, preserved in formalin, flash frozen, or used to establish other preclinical models (Figure 10). Thus, maintaining accurate records of tumor material storage is paramount. The biobank database should not only contain searchable information about the number, type, and storage location of aliquots, but also records of sample usage and transfer to other investigators.

### Histology, Genomic, and Preclinical Data Annotation

As discussed above, comparison between genomic and histology profiles of patients and PDX samples is essential to determine whether the PDX indeed recapitulates the main characteristics of the corresponding human disease. Whenever possible, high-resolution representative histology slides detailing morphology of the patient and PDX tumors, and corresponding pathology reports should be uploaded in the database. Similarly, the database should integrate genomic data that may have been captured through multiple platforms for both patient (tumor and normal) and corresponding PDX samples to facilitate comparative cross-species and longitudinal analyses (5) (Figure 9). Finally, the results of any preclinical and co-clinical trials conducted with established PDX models should also be uploaded in the database, as such data can help inform the planning of further translational studies.

Overall, the goal of assembling a comprehensive PDX database is to provide investigators and administrators with a coherent presentation of all their models, allowing them to better utilize their valuable resources. Importantly, access to this resource should be regulated so only authorized personnel have access to clinical information, while other users can only access de-identified data sets, although will still be able to query the database for information on availability of specific PDX models based on tumor subtype, defined genetic alteration, resistance to targeted therapy, or other criteria. Access to this plethora of well-organized information in the end will foster collaborations and, ultimately, scientific advances.

Costs associated with establishing and maintaining PDX libraries, even in the context of centralized PDX programs, are quite substantial. These costs can vary significantly based on level of institutional support, available infrastructure, local cost of labor and reagents, and extent of characterization of each PDXs model.

**Figure 11:**
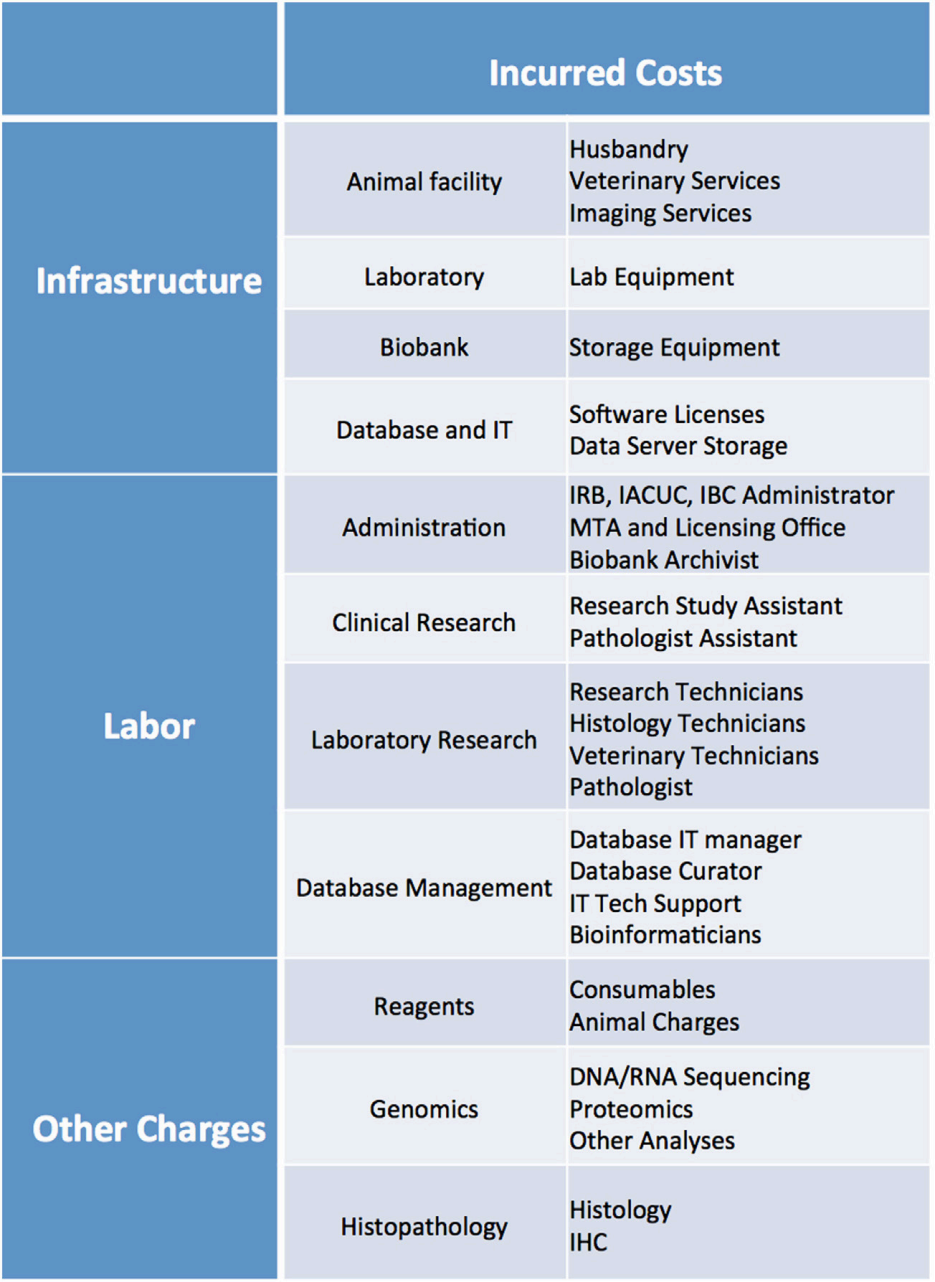
List of infrastructure, labor, and reagent costs likely to be incurred when establishing PDX models. IRB, Institutional Review Board; IACUC, Institutional Animal Care and Use Committee; IBC, Institutional Biosafety Committee; MTA, Material Transfer Agreement; IT, Information Technology; IHC, Immunohistochemistry.

Cost analysis should take into account several parameters, including: cost of reagents (consumables, mice, sequencing and histology costs, etc.), cost of infrastructure (animal facility husbandry and veterinary costs, biobank storage, animal imaging services, database maintenance and data server charges, software licenses, laboratory equipment), and cost of labor (research techs, veterinary staff, clinical research assistants, histology techs, administrative staff, database managers, bioinformaticians, IT support) (Figure 11). While more thorough cost analysis considerations can be found in Krivtsov et al. (5), in our experience, the average cost of establishing a PDX model is ~$1,500–2,000, excluding costs associated with the model’s genomic and histology characterization.

## PDX Use in Preclinical Studies

Patient-derived xenograft models are often preferred to cell xenograft models and Genetically Modified Mouse (GEMM) models as preclinical cancer models because they more closely recapitulate human tumor heterogeneity and have the potential to better reflect the range of mutations and genetic background present in patient populations. For these reasons, they are often considered better predictors of treatment response (61, 62).

In the case of studies aimed at validating efficacy of targeted therapeutics, trials can be conducted on multiple PDX models harboring mutations in the same pathway, and are often compared to response in GEMMs, and cell xenografts (63). When extensively genomically annotated PDXs of the same tumor type are abundant, they can also be employed in large-scale studies to correlate genomic profiles to treatment response, and to facilitate discovery of novel biomarkers (64, 65).

In addition, PDXs can be used to investigate primary and acquired resistance to therapy. This entails collecting tumor tissue from the same patients at different stages of their disease, before therapy and then at time of relapse. This is not always a feasible strategy, especially for tumor types in which surgical procedures are not part of the standard of care at the time of disease progression. Thus, investigators can also use PDX models established from treatment-naïve tumors, and expose them to multiple cycles of treatment, until they develop resistance (66, 67). In cases of patients with metastatic tumors resistant to multiple lines of therapies, PDXs can be generated from different lesions, which may have acquired resistance through different pathways (68). In such cases, tumor samples may also be collected at time of death, from patients that had enrolled in a research autopsy program (69). In general, PDX models are not intended to help inform clinical decisions on the patient they are derived from, mainly because cancer treatment strategy may need to be implemented in the patient well before the PDX model has engrafted. However, several successful cases of personalized treatment have been reported, and clinical trials are in progress to determine if this is indeed a valid approach in ovarian cancer, sarcomas, and other cancer types (62).

Execution of preclinical studies in PDX models is often challenging, because of logistical, technical, and financial hurdles. In many instances, this effort is beyond the capabilities of individual investigators. To obviate to this issue, institutions heavily committed to translational research and precision medicine may opt to establish a centralized PDX Core (5) and Mouse Hospital (70) to manage their PDX Program. In such cases, patient sample screening, collection, processing and implantation, along with management of mouse colonies, transplantation of established PDX models, and general maintenance of PDX libraries and data management and integration, is all performed through the concerted efforts of a specialized PDX team.

There are several benefits associated with implementation of such programs. By utilizing standard procedures and workflows, patient sample collection and implantation can occur in a concerted fashion, significantly reducing ischemia time and thus improving chances of tumor engraftment. Additionally, the employment of specialized technicians for all procedures allows for efficiency and accuracy. Finally, integrated data management, from PDX clinical and genomic annotation to biobanking records, allows PDX users to maximize the utility of the PDX models, and fosters establishment of collaborative projects.

In this setting, within the Mouse Hospital, enrollment of PDXs in preclinical trials is overseen by specialized PDX technicians familiar with the characteristics of each disease model and well versed in all technical aspects of tumor transplantation, drug administration, and both clinical and efficacy assessments. Studies are conducted following detailed SOPs for all procedures. Once again, this ensures study accuracy and reproducibility, and the centralized operation helps contain costs.

In other instances, PDX programs can be established through collaborative networks among academic institutions with the goal of creating large-scale PDX platforms through which patient specimens and PDXs, along with their clinical annotation, can be efficiently shared (62). In these instances, standardization of methodologies, harmonization of clinical annotations, and genomic data among different institutions may present several challenges, and extensive inter-institutional administrative and IT support is essential.

## Conclusions

Patient-derived xenograft models have become a highly desirable platform in oncology and are expected to substantially broaden the way *in vivo* studies are designed and executed and to reshape drug discovery programs. They represent an invaluable tool for a number of applications, including tumor genetics, biomarker discovery, the study of metastatic progression, the fate of CTCs, and the development of novel therapies for early, advanced, and drug-resistant tumors. For large institutions, a centralized PDX core capable of combining existing resources with new infrastructure to create an integrated organization can be a solution that is both cost-effective and efficient. This in turn can lead to an increase in PDX library size, better use of the established PDX models, proliferation of collaborative initiatives, and ultimately development of more knowledge to advance cancer medicine.

## Author Contributions

MM, CM, AK, BQ, and SG contributed written protocols, figures and tables. EdS reviewed and revised all written material.

## Conflict of Interest Statement

The authors declare that the research was conducted in the absence of any commercial or financial relationships that could be construed as a potential conflict of interest.

## Footnotes

^1^www.hhs.gov/ohrp/regulations-and-policy/regulations/index.html.

^2^www.hhs.gov/hipaa.

^3^https://www.osha.gov/SLTC/bloodbornepathogens/standards.html.
